# Ways of coping with excessive academic stress among Korean adolescents during leisure time

**DOI:** 10.1080/17482631.2018.1505397

**Published:** 2018-08-12

**Authors:** Se-Hyuk Park, Youngshim Kim

**Affiliations:** a Department of Sport Sciences, Seoul National University of Science and Technology, Seoul, Korea; b Department of Early Childhood Education, Soongsil Cyber University, Seoul, South Korea

**Keywords:** Stress-coping strategies, academic stress, adolescents; leisure; stress-related growth

## Abstract

**Purpose:** Korean adolescents are under excessive academic pressure because of the competitive college entrance examination. This study examined the characteristics of coping strategies on the academic stress experienced by senior high school students in Korea. **Methods:** A total of 11 adolescents who were preparing for the college entrance examination participated in this study. This study employed semi-structured in-depth interviews. A constructive grounded theory was employed to capture the characteristics of stress-coping strategies among adolescents. **Results:** After analyzing the data, we identified four themes as strategic attributes of stress-coping for academic stress among Korean adolescents: (a) creating coping strategies in a physically active manner; (b) creating coping strategies in a non-physical and positive form; (c) utilizing maladaptive coping mechanisms; and (d) relying upon religious belief and spiritual power. **Conclusion:** This study indicates that Korean adolescents developed their own coping strategies to deal with various academic stressors through either positive or negative forms of leisure.

## I. Introduction

A great deal of the literature and research dealing with stress during adolescence has cited school as a major contributor to adolescents’ stress. Empirical studies have demonstrated that academic issues are generally a main concern of adolescents around the world, and the excessive academic pressure to perform better in schoolwork is more acute in South Korea. There is a visible increase in the incidence of psychological problems leading to stress and the effects of stress for Korean adolescents (Ha, 2015). Education is highly valued in Korea, and the expectations of parents, teachers, and students themselves to excel academically can also be a source of intense stress for many students. Therefore, the extreme performance pressure placed on high school students has been subject to criticism (Demircan & Demir, 2014; Kim & Lee, 2013; Oktan, 2014).

The academic performance of Korean adolescents is important for their psychosocial development and to prepare them for entering a college as well as adulthood. High school students in Korea attend classes from approximately 08:00 to 17:00. Nearly all Korean high school students also go to private after-school institutions or stay at school for evening individual study that may continue until 22:00. Under strong sociocultural and psychological influences emphasizing the importance of college entrance examinations, Korean adolescents further curtail their sleeping time (Bae & Wickrama, 2015). In addition, the quality and amount of parental involvement and investment in children’s academic performance seem to cause excessive stress for adolescents (Kim & Lee, 2013).

Academic stress is known to be deleterious to adolescents’ health by promoting maladaptive behavioural coping responses such as smoking and alcohol intake (Glozah & Pevalin, 2014). In empirical research, academic stress has been widely linked to several outcomes such as adjustment, mental health, suicidal intention, and academic achievement (Park, Kwon, & Shin, 2014). Coping with stress is one of the topics that has been most widely studied in contemporary psychology. Park, Kwon, et al. (2014 ) indicated that active coping fully mediated the relationship between academic stress and school adjustment for gifted high school students. Evidence has also been found that coping influences the relationship between stress and illness or health (Kim, Kim, & Jung, 2014; Lazarus, 1999).

Until now, very limited studies have examined the relationship between students’ academic stress and their coping strategies, especially in leisure time. Coleman (1993) argued that leisure activities are thought to play a buffering role against the effects of stress. Especially, leisure participation has been shown to be associated with psychological well-being and academic performance (Bartko & Eccles, 2003; Trainor, Delfabbro, Anderson, & Winefield, 2009). It has also been noted that personality and individuals’ life circumstances influence the ways in which they use leisure to cope with stress (Zuzanek, Robinson, & Iwasaki, 1998). That is, utilization of leisure may be one coping strategy for academic stress for adolescents.

Generally leisure is regarded as a positive force, facilitating the realization of a wide variety of physical, social, emotional, and psychological developments for adolescents. On the other hand, it is also a time that goes against socially acceptable and conventional norms such as vandalism, bullying, sexual or substance abuse, running away, violence, substance misuse, and other forms of delinquent activities (Mahoney & Stattin, 2000). Stebbins (1996) conceptualized deviant leisure as behaviour that violates criminal and non-criminal moral norms. According to Rojek (1999), the terms of deviant leisure, leisure, and crime all share similar attributes, and they can be referred to differently depending upon particular cultural and social contexts. Wang, Chen, Lin, and Wang (2008) proposed that leisure may provide a context in which adolescents are engaged in delinquent behaviours as well as self-developmental activities. In this regard, several researchers have suggested that engagement in delinquent activities may be considered as leisure (Verkooijen, Nielsen, & Kremers, 2008).

It can be assumed that Korean adolescents may use their leisure time to cope with excessive academic stress in various ways, either positive or negative. Therefore, this study attempted to identify how Korean adolescents cope with excessive academic stress during leisure time. This study’s research question was: what leisure strategies do students employ to overcome academic stress?

## II. Method

This study employed semi-structured in-depth interviews. A constructive grounded theory was employed to capture the characteristics of stress-coping strategies among Korean adolescents. We attempted to formulate a constructive grounded theory in which “discovered reality arises from the interactive process and its temporal, cultural, and structural contexts” (Charmaz, 2000, p. 524). By utilizing this method, we could explore deeper understanding and participants’ experience of a particular social context. Constant comparative analysis was employed under a structure of Charmaz’s analytical approach in creating emerging theory (Charmaz, 2006). By using this methodology, we facilitated exploration of deeper understanding and analysis of adolescents’ stress-coping experience during leisure time in relation to the competitive college entrance examination. In addition, we used a back-translation method based on previous studies (Suh, Kagan, & Strumpf, 2009).

### Participants

For this study, we selected Korean senior high school students as a sample who had prepared for the college entrance examination. A total of 11 adolescents, who were preparing for the college entrance examination, participated in this study (eight females and three males). Their ages ranged from 17 to 18 years. Out of the 11 participants, six were Christian, two belonged to Buddhism, and the others did not have a religion. Among them, two students had stayed at a school dormitory.

### Data collection procedure

Both purposeful and snowball sampling were used to recruit participants for this study. The researchers received permission from participants’ parents for them to participate in this study. We provided a brief introduction of the study’s purpose before the interview. The researchers provided the participants with information regarding confidentiality. We also explained that the participants were under no obligation to complete their participation. The participants voluntarily engaged in this research study and only pseudonyms were used for identification. Interviews were conducted at locations that were most convenient for the participants. Interviews were conducted at a café, park, or on the phone depending on the situation. The interviews lasted between 45 and 75 minutes, and were tape recorded and transcribed with the participants’ permission. Extensive ethical attention was given to how researchers protect participants during data collection. Opportunities were provided for participants to reply and liaise with us during this process. Before conducting interviews, we clearly identified the relevance and value of the method within our design.

As proposed by Legard, Keegan, and Ward (2003), we used content mapping and content mining questions. The content mapping questions allowed us to widen the participants’ perspectives, stimulate thoughts and experiences, and ensure comprehensive coverage. Among the content mining questions, amplificatory probes and clarification probes enabled us to obtain detailed and clear descriptions of academic stress related experiences (Legard et al., 2003). Follow-up probing questions were also used to elicit richer and more in-depth responses from interviewees (Patton, 2002). Field notes were taken during the interviews. Notes were also taken to assist with the transcribing processes. The following are a few examples of the questions asked: “Do you experience academic stress in preparing for the college entrance examination?” “How do you deal with the stress during leisure time?” “What benefits do you experience when you participate in the leisure time activity?” The number of interviews was decided based on the principle of data saturation (Guest, Bunce, & Johnson, 2006). Interviews were conducted and coded until no new themes or categories emerged.

### Data analysis

To obtain emerging themes and categories across the data, we utilized the constant comparative method, which allows researchers to simultaneously code and analyze data to develop concepts and to compare interviewees with one another (Kim, Park, Malonebeach, & Heo, 2016; McCracken, 1988). Two investigators coded the transcripts, and made comparisons prior to finalizing the codes. The researchers followed the four-step method of data analysis (Patton, 2002). Step 1: we analyzed raw data to extract important ideas from the transcripts. Step 2: we compared transcripts to determine whether preliminary categories were consistently classified. Step 3: we developed clusters of ideas related to the preliminary codes. Step 4: finally, we delineated the core themes and selected data examples to support those themes. Using quotes and illustrative examples of each theme, we came up with a consensus. Once the transcripts had been coded using the framework, quotes for each code were grouped together and summarized, and specific quotes were chosen that best represented the themes outlined in the coding framework, and a selection of these were presented as part of the results.

This study employed two strategies to increase the credibility of the data: an expert review process and a member-checking process (Creswell, 2009; Kim et al., 2016). First, using the expert review process, we conducted peer-debriefing processes with colleagues, providing opportunities for us to challenge each other’s interpretations and improve the credibility of the data. Second, we contacted the participants and explained a summary of the identified core themes and interpretations. Participants were asked to review the interview transcripts to verify the accuracy of the analysis. Of the 11 participants, eight participants voluntarily participated in the member-checking process and expressed their satisfaction with the interpretations. We additionally conducted both member-check interview and member-check focus group methods for confirmation, modification, and verification of the interview transcript. According to Harvey (2015), the member-check interview is congruent with the epistemology of constructivism because undertaking a second interview to discuss data empowers participants to remove and add to their data thereby co-constructing new meanings. In addition, the member-check focus group method was utilized in a cafe for 1 hour to explore the opinions and behaviours of a group of four participants to respond and interact together, enhancing the trustworthiness of the results.

## III. Results

After analyzing the data, we identified four themes as strategic attributes of stress-coping for academic stress among Korean adolescents: (a) creating coping strategies in a physically active manner (e.g., sport club activities and jogging); (b) creating coping strategies in an inactive/positive manner (e.g., listening to music, internet surfing, and writing an essay); (c) utilizing maladaptive coping mechanisms (e.g., overuse of internet games and/or socially deviant behaviours); and (d) relying upon religious belief and spiritual power (e.g., prayer and bible study activities). This study indicates that Korean adolescents developed their own coping strategies to deal with various academic stressors. It also suggests that a challenging and stressful life circumstance in relation to academic stress may provide opportunities for Korean adolescents to increase their inner strengths and potentials as well as to cause emotional and psychological problems. This study indicates that Korean adolescents developed their own coping strategies to deal with various stressors through the use of either positive or negative forms of leisure.

### Creating coping strategies in a physically active manner

Physically active leisure has been defined as activities that encompass exercise and sport activities such as jogging, swimming, and cycling. It is well documented that physical activity is an important contributor to the prevention and reduction of academic stress (Aaltonen, Latvala, Kujala, Kaprio, & Silventoinen, 2016; Park, 2014a). Wunsch, Kasten, and Fuchs (2017) reported that physical activity in the academic examination period may be able to buffer the negative impacts of stress on health-related consequences. That is, sport and exercise can be used for preventive and therapeutic purposes for adolescents facing academic stress. In addition, links between leisure or sport and lowered delinquency or deviant behaviours among adolescents have been reported (Miller, Sabo, Farrell, Barnes, & Melnick, 1998; Nichols, 2004; Persson, Kerr, & Stattin, 2007). However, the majority of Korean adolescents are not physically active enough to achieve health benefits mainly due to the excessive school work (Ha, 2015). The majority of participants were positive about participation in exercise and sport activities. Additionally, three participants expressed the view that when they participated in physically active leisure activities, they were more likely to sleep well, less likely to experience excessive stress while studying, and less likely to be a problematic internet user. For example, Mathew (male 18) stated:

For me, playing basketball game by myself is fun and stress releasing. Dribbling and shooting make me sweaty and exciting. Sometimes, I unconsciously dribble and shoot them. Especially, when I make a goal, it is really exhilarating. Sometimes, I play basketball games with others … When I bump into other guys in the game, I feel vitality and toughness.

It appears that they became mentally and physically stronger and increased their resilience to academic stress by engaging in physical activities. It was also pointed out that the lack of recreational programmes in close proximity to where they lived was a major constraining factor in engaging in physical activities in their leisure time. It appears that attractive and reasonable school or community sponsored exercise programmes may attract adolescents to do exercise. It is clear that accessibility of facilities or programmes is critical for adolescents to engage in physical activities. Doris (female, 18 years) mentioned:

Because I stay in the school dormitory, I can’t frequently participate in exercise programmes. My school needs to provide various kinds of exercise programmes after school. I like to participate in a boxing aerobics programme. It is close to my house. I could participate in the programme 2–3 times a week during summer vacation. However, I could do it once a week because of time constraint during semesters. When I got sweaty and my clothes got wet, I felt that I became healthy …. When I shout and scream in the boxing aerobics programme, all the pains went away. I could also reduce weight …. I can escape from bad school life.

According to nine participants, actively singing and dancing at “Noraebang”, in which singing audio-visual systems and space are provided, reduces their level of academic stress. Mary (female, 18 years) said that she normally goes to Noraebang with friends after mid-term or final examinations. She mentioned that they could scream and shout while singing and dancing, resulting in stress reduction.

### Creating coping strategies in a non-physical and positive form

Employing non-physical and positive coping strategies was the most salient theme that emerged from the data. These leisure activities included listening to music, chatting, internet surfing, playing smartphone games, and writing an essay. By engaging in such activities, they could deal with various academic stresses in a relaxed manner. The vast majority of sport-related research on the effects of inactive leisure activities, such as internet games, has been focused on the negative impact. Several researchers contended that well developed and guided inactive leisure activities may also have the potential to enhance mental health and well-being in adolescents (Granic, Lobel, & Engels, 2014; Park, 2014b). Most of the participants responded that they enjoy chatting with friends. In particular, eight participants preferred to chat on the phone. For example, Mary (female, 18 years) said that she would write an essay when she was faced with stress. According to all of the participants, listening to music helped them reduce their level of academic stress. For example, Catherine (female, 17 years) said:

I like to listen to music while I study. It makes me feel comfortable. Like me, most of the students studying at school library listen to music when they are studying. I prefer to listen to classic or popular songs. Sometimes, I listen to pop songs and light music. However, I like to listen to rap music when I want to totally relax during free time. Especially, I like BY’s (one of the most popular rap musicians in Korea) rap music. I may say that his rap music is really healing to me.

Nine participants indicated that engagement in internet surfing and internet games provided rich opportunities to escape from academic stress. For instance, Linda (18 years) stated that she mainly engaged in internet surfing to find information concerning entertainers and idol singers. It appears that internet surfing or games make the respondents feel refreshed and relaxed. She also expressed that she often felt irritated due to having academic work to do while she was enjoying internet surfing and games. Mathew (male, 18) expressed the point that staying alone and watching movies in his room made him peaceful and calmer. He further stated in relation to internet games:

I enjoy playing sea of the storm which is related to destroying opponents’ buildings. Although the game is not popular among my peers, I enjoy it. I feel competent while I am playing the game. I escape from academic stress for a while at least. Although I still recognize the reality of academic pressure, it is great to release stress even for a second. Actually, I want to be a professional gamer in the future.

### Utilizing maladaptive coping mechanisms

Another theme, which was coming up very strongly in the interviews, is the utilization of maladaptive coping mechanisms. Although leisure is generally believed to have positive benefits to adolescents, it also has many qualities in common with delinquency. Wang et al. (2008) indicated that leisure time may provide a context in which adolescents are engaged in delinquent or violent behaviours as well as self-developmental activities. It should also be recognized that deviant and negative leisure engagement has been linked with academic stress, causing vandalism, bullying, smoking, drinking, and undesirable activities for adolescents. Megan (female, 18) pointed out that unstructured leisure time may lead to deviant or violent behaviours for adolescents. In addition, despite the benefits of internet use, excessive internet usage negatively affects adolescents’ growth and development. Shin and So (2012) reported that Korean adolescents who spend more time in physical inactivity and online gaming are likely to receive a below-average academic record. Several participants indicated that as they get more addicted to internet and smartphone games, they become more engaged in deviant activities during their leisure time. Unfortunately, the internet and smartphones are major tools for information, communication, and entertainment in the daily lives of Korean adolescents. For example, Peter (male, 17 years) said:

Sometimes, I am afraid of staying alone … I play aggressive internet games to release stress. It makes me feel good. Often I enjoy watching pornography through smartphone and internet at internet café. When meet my friends at internet café, we get together and feel camaraderie …. We smoke and drink together.

Bullying, violence, vandalism, and drinking can be regarded as a maladaptive stress-coping mechanism. Espelage and Swearer (2003) contended that bullying can be viewed as a factor that helps adolescents attain dominance in newly formed peer groups. Neal (2010) argued that adolescents tend to organize themselves in social hierarchies and compete for access to their peers with both cooperative and coercive strategies to advance in the peer social hierarchy as well as to gain peer admiration. This is related to aggressive behaviours in which an individual or a group repeatedly conduct negative or bothering actions against individuals who are not able to defend themselves. John (male, 17 years) said that he enjoys teasing his brother and friends. He believed that such behaviour was just playing, and that he was trying to warn them of their socially awkward behaviours. He further said:

One day, I was with a friend of mine in a playground. He was very quiet and … didn’t talk much. I just didn’t feel good and started kicking him for no reason. Sometimes, I tried to change my character, to become cool. But, it doesn’t work. I don’t know why I behave like that. To tell you the truth, what really annoys me is the fact that teachers, friends, and parents treat me like a criminal. Well … Sometimes, I break others’ private equipment or public facilities with my friends for fun.

Lloyd, Tafoya, and Merritt (2015) contended that deviant behaviours such as drinking alcohol during leisure time may contribute to binding adolescents together through a sense of group membership. Drinking alcohol may be considered by adolescents as a cheaper and more attractive way to enjoy leisure time with peers. Among adolescents, drinking with peers may be considered as a medium to identify themselves and help them escape from academic stress. In addition, drinking alcohol may provide adolescents with opportunities to enjoy freedom and to escape from their daily lives. The following quote illustrates the contribution of drinking (Peter, 17 years):

Well, I like just drinking and having a laugh … Most of my friends drink alcohol as I do. Just losing consciousness for a couple of hours makes me feel like a bird. I just forget academic stress and family problems … Um, everything just seems to go away.

### Relying upon religious belief and spiritual power

Participation in religious activity has been linked to outcomes such as better perceived health, decreased aggression, increased self-esteem, less suicidal ideation, and lower prevalence of depression (e.g., Bremner, Koole, & Bushman, 2011; Crescentini & Capurso, 2015; Koole, Govorun, Cheng, & Gallucci, 2009). It is interesting that some of the participants who are Christian responded that they find strength and peace from prayer meetings and meditation in their daily lives. Five participants were involved with various forms of spiritual activities such as prayer and bible study activities, and, as a result, considered themselves to be better able to cope with academic stress. The most commonly described religious and spiritual activity used to manage excessive academic stress was prayer. Participants said they mainly prayed alone. Prayer reportedly allowed participants to ask for help and to help get them through academic challenges. The following quote illustrates the contribution of prayer (Sony, female, 17 years):

I could not make it through. I can’t even make it through a day without prayers. Whatever crosses my way, I give thanks for them. And I give thanks for everything for the good and for the bad.

Linda (female, 18 years) explained that she goes to a church with friends and develops intimate friendships, through which she reduced academic stress and depression. She further indicated that she regularly participates in chapel offered at school, providing her energy and psychological well-being. For example, Sony (female, 18 years) mentioned:

I feel rejuvenated when I go to church. The pastor for our Sunday school gives us messages which are positive and encouraging. Through participation in Sunday school service, I often become confident in preparing for college entrance examination …. When I am depressed in school, I sometimes make a phone call to the pastor to be equipped with spirituality. And often I listen to gospel songs to overcome depression and gain spirituality during leisure time.

It appears that such positive interactions with friends through religious activities helped the participants to reduce their stress. For example, Susan (female, 18 years) mentioned that sharing her spirituality with others helped her to enhance her inner peace and stress management skills. She further indicated that when they attend fellowships and participate in a bible study, they felt they ameliorate their stress and increase their psychological well-being as well as spirituality. Sony (female, 18 years) also indicated that she came to grow as she overcame academic stress in Jesus. Similarly, Linda (female, 18) expressed thanks to the Lord for having difficulty, which made her renewed. Tari (female, 18 years) expressed a view that a spiritual relationship with friends gives her power and consolation. Tari stated:

I often find good friends in the bible study. When I talk with friends in the bible study, my anger and aggression levels come down. I feel better. I feel thankful to the Lord. After the bible meeting, I can concentrate on studying more effectively.

## IV. Discussion

There exists a strong culture of social expectation pertaining to high academic achievement in South Korea. Thus, Korean adolescents are under excessive academic pressure because of the competitive college entrance examination. Our results strongly support the assertion that leisure may provide an opportunity to restore the disruption of adolescents’ normal life patterns when they experience academic stress. That is, participation in recreational activities is related to the effective management of major life stressors for adolescents. A few researchers have emphasized the value of a problem-solving coping mechanism among Korean adolescents (e.g., Park et al., 2014). They considered a problem-solving coping style to be an active coping mechanism. According to D’Zurilla and Sheedy (1992), the more the adolescents demonstrated problem-solving coping skills, the less they perceived academic stress.

The present study indicated that adolescents who utilized more problem-solving coping skills reported higher levels of psychological adjustment. In the present study, we mainly proposed two categories of academic stress-coping response: positive problem solving and maladaptive coping strategies. While positive problem solving involves working on a problem and remaining healthy, maladaptive stress coping is related to engagement in deviant and/or negative leisure activities. Constructivism can be regarded as the most critical feature in dealing with excessive stress or trauma to achieve higher levels of psychological adjustment (Neimeyer & Young-Eisendrath, 2015). Constructivism stresses an individual as a scientist who actively learns to conduct his or her own life through personal experiences and builds the world in which he or she lives (Alves, Mendes, Goncalves, & Neimeyer, 2012). Neimeyer proposed that human beings do not merely react to the world, but rather act on it, even making the stressful event generate growth. On the other hand, it is also possible that adolescents who are exposed to excessive academic stress are likely to seek ways to release excessive stress through deviant forms of leisure. Thus, it is critical to provide adolescents with attractive, educational, and easily accessible recreation programmes both at schools and in communities. In addition, stress management programmes need to be developed specifically for adolescents to effectively and constructively deal with academic stressors. The current study supports the previous findings that adolescents utilize maladaptive coping mechanisms such as dissociation, isolation, and violent behaviours to deal with conflicts (Abdukeram, Mamat, Luo, & Wu, 2015; Oh & Kim, 2014; Windle & Mason, 2004; You, Kim, & Kim, 2014).

Several scholars (Greenfield & Yan, 2006; Park, Kang, Kim, 2014) have indicated that excessive internet usage negatively affects adolescents’ growth and development. In line with Donati, Chiesi, Ammannato, Primi (2015), this study demonstrated that excessive internet use could result in addictive behaviours and problematic internet use, which “creates psychological, social, school, and work difficulties in a person’s life” (Beard & Wolf, 2001, p. 378). Previous studies commonly also revealed that psychopathologies such as academic stress affect problematic internet use in adolescents (e. g., Park et al., 2014). The current study also noted a positive association between excessive internet use in leisure time and delinquency for adolescents. Further, it can be assumed that deep absorption in online gaming and smartphone use may result in low academic performance.

Prior studies provided evidence that an active leisure life, as opposed to a passive or sedentary leisure lifestyle, has a positive effect on coping with stress and maintaining good health. The findings of this study are aligned with previous studies in that adolescents who participated in physical activities demonstrated lower levels of academic stress than those who are involved in inactive/passive leisure activities such as excessive computer and smartphone games. Korean adolescents experienced a psychological comfort zone and psychological well-being through participation in physically active leisure activities. Consistent with Park’s (2014a) study, this study suggests that physically active adolescents were more likely to express satisfaction with their sleep, less likely to feel stress in their lives, and less likely to be a problematic internet user compared to physically inactive adolescents. The findings of this study are also consistent with previous studies that a negative relationship exists between sport participation and delinquent behaviour among adolescents (Miller et al., 1998). These findings are consistent with Park’s (2014b) view that adolescents’ deviant behaviours can be reduced by having goal-oriented recreational sport activities through which to learn to overcome academic pressure, and to become more aware of healthy leisure opportunities.

Researchers have given only scant attention to the potential of non-physical forms of leisure to provide stress-coping benefits (Caltabiano, 1994; Park, 2015). That is, non-physical forms of leisure such as internet surfing and listening to music in a healthy manner may contribute to lessening academic stress. Thus, more research is needed to understand better the role of non-physical forms of leisure, including social and physically inactive leisure activities, in coping with academic stress. The present study highlights the positive side of non-physical forms of leisure activities such as internet surfing, chatting, and listening to music. It should be noted that the proper use of the internet also contributes to adolescents’ health, while excessive internet use causes psychological, physical, and social problems for adolescents.

It can be also proposed that spiritual experiences could occur in a range of leisure contexts. Heintzman (2002) identified themes indicating that leisure time could provide a time and space for spiritual development. In this regard, leisure can also be identified as a context in which an individual can be inspired to spiritual values.

The spiritual core is the place of our soul, unlimited in its expansiveness. Spirituality impels us to seek and to discover the more of who we are and calls us to enter the depths of our being, where we discover our intrinsic connectedness with all of life and with the external oneness and sacred source of our being. (Burkhardt & Nagai-Jacobson, 2002, pp. 3–4)

While these findings help reaffirm previous research and conceptual discussions of the spiritual potential of leisure, the relationship between the spiritual domain and leisure needs to be further studied. It was evident that participation in religious activities by adolescents is linked to outcomes such as increased subjective well-being, decreased depression, higher life-satisfaction, and reduced academic stress.

Overall, the findings from this study highlight the importance of paying attention to the different types of leisure participation as a means of coping with academic stress and maintaining good health for adolescents. It is critical to provide adolescents with opportunities for a healthy leisure environment and with programmes to effectively face academic stress. It can be further suggested that a challenging and stressful life circumstance in relation to academic pressure may provide opportunities for Korean adolescents to experience stress-related growth as well as emotional and psychological problems.
